# Endometriosis and ART: A prior history of surgery for OMA is associated with a poor ovarian response to hyperstimulation

**DOI:** 10.1371/journal.pone.0202399

**Published:** 2018-08-20

**Authors:** Mathilde Bourdon, Jade Raad, Yaniv Dahan, Louis Marcellin, Chloé Maignien, Marc Even, Khaled Pocate-Cheriet, Marie Charlotte Lamau, Pietro Santulli, Charles Chapron

**Affiliations:** 1 Department of Gynecology Obstetrics II and Reproductive Medicine; Université Paris Descartes, Sorbonne Paris Cité, Centre Hospitalier Universitaire (CHU) Cochin, Paris France; 2 Department “Development, Reproduction and Cancer”, Institut Cochin, INSERM U1016, Université Paris Descartes, Sorbonne Paris Cité, Paris, France; 3 Department of Histology-Embryology and Reproductive Biology; Université Paris Descartes, Sorbonne Paris Cité, Centre Hospitalier Universitaire (CHU) Cochin, Paris, France; Seoul National University Bundang Hospital, REPUBLIC OF KOREA

## Abstract

**Background:**

Many women whose fertility may have been impaired by endometriosis require assisted reproductive technology (ART) in order to become pregnant. However, the influence of ovarian endometriosis (OMA) on ovarian responsiveness to hyperstimulation has not been clearly established.

**Objective:**

To evaluate the risk of a poor ovarian response (POR) to stimulation and ART outcomes in women with OMA.

**Materials and methods:**

We conducted a large observational controlled matched cohort study in a tertiary care university hospital between 01/10/2012 and 31/12/2015. After matching by age and anti-Müllerian hormone (AMH) levels, 201 infertile women afflicted with OMA (the OMA group) and 402 disease-free women (the control group) undergoing an ART procedure were included in the study. The main outcomes that we measured were a POR to hyperstimulation (i.e., ≤ 3 oocytes retrieved, or cancelled cycles), the clinical pregnancy rate, and the live birth rate.

All of the women with endometriosis underwent a pre-ART work-up, in order to obtain an accurate diagnosis and staging of their disease. An OMA diagnosis was based on published imaging criteria (obtained by transvaginal sonography or magnetic resonance imaging) or on histological analysis for patients with a prior history of endometriosis surgery. The statistical analyses were conducted using univariate and multivariate logistic regression models.

**Results:**

The incidence of a POR to hyperstimulation was significantly higher for the OMA group than for the control group [62/201 (30.8%) versus 90/402 (22.3%), respectively; p = 0.02]. However, no significant differences were found between the OMA and the control group in terms of the clinical pregnancy rate [53/151 (35%) versus 134/324 (41.3%), respectively; p = 0.23] and the live birth rate [39/151 (25.8%) versus 99/324 (30.5%), respectively; p = 0.33]. By multivariate analysis, a prior history of surgery for OMA was found to be an independent factor associated with a POR to stimulation [OR = 2.1; 95% CI: 1.1–4.0], unlike OMA without a prior history of surgery [OR: 1.5; 95% CI: 0.9–2.2].

**Conclusion:**

The presence of OMA during ART treatment increased the risk of a POR to hyperstimulation, although the live birth rate was not affected. Furthermore, having OMA and having previously undergone surgery for OMA was identified as an independent risk factor for a POR.

## Introduction

Endometriosis is a chronic and painful disease caused by the growth of endometrial-like tissue outside of the uterus that generally induces a chronic inflammatory reaction and infertility [1]. It is a disease that is heterogeneous in nature, with lesions exhibiting three distinct phenotypes [2]: (i) superficial peritoneal endometriosis (SUP), (ii) ovarian endometrioma (OMA), and (iii) deeply infiltrating endometriosis (DIE).

OMA, which tends to be the most common manifestation of endometriosis, is often associated with pelvic pain and infertility [2]. It occurs in up to 20–40% of women with endometriosis who are undergoing in vitro fertilization (IVF) [3]. The presence of endometrioma has been reported to have a detrimental impact on ovarian responsiveness to hyperstimulation [4,5]. However, despite an abundant literature on OMA-related infertility, the mechanisms by which it impairs ovarian stimulation are still unclear. In a recent systematic review, Hamdan *et al*. concluded that women with non-operated OMA undergoing In Vitro Fertilization/ Intra Cytoplasmic Sperm Injection (IVF/ICSI) had a significantly lower number of retrieved oocytes, the cycle cancellation rate was significantly higher compared with those without the disease, and surgery for the OMA did not reduce the ovarian response. The authors therefore postulated that it was likely that the reduced ovarian response was due to the presence of the OMA itself [4]. On the other hand, Somigliana *et al*. identified seven studies evaluating ovarian responsiveness to hyperstimulation in non-operated women with unilateral OMA [5]. These authors concluded that the response rates of the affected and the contralateral ovary were similar [5]. There are only a small number of studies in the literature of the impact of bilateral non-operated OMAs on oocyte quality and pregnancy outcomes. These studies did not find any evidence of detrimental effects of non-operated bilateral OMAs on pregnancy rates, although the small sample sizes could have skewed the results [6,7].

Although this topic has been the subject of extensive investigation and debate, a credible explanation for the discrepancies between several of the studies is still lacking [3–5]. Thus, the specific impact of OMA itself, the influence of an associated deep endometriosis phenotype, and the impact of surgical treatment for OMA on ovarian responsiveness to hyperstimulation are aspects that require further elucidation. Additionally, in numerous studies on this topic, the level of anti-Müllerian hormone (AMH) was not taken into account. There is, however, a general consensus that AMH levels predict the magnitude of the controlled ovarian stimulation (COS) response [8]. Indeed, serum AMH levels have proven to be a reliable surrogate marker of the ovarian reserve, with an established correlation with age, antral follicle count, and the response to controlled ovarian hyperstimulation [9,10]. Discrepancies among the studies to date could be related to significant confounders, such as the variation of the ovarian reserve associated with endometriosis in particular. In the context of OMA-disease, in order to overcome potential bias related to ovarian reserve disparity, we assessed the impact of OMA on the ovarian responsiveness to hyperstimulation and ART outcomes in this observational controlled cohort study. To do so, we analyzed a consecutive series of women with OMA and disease-free counterparts who had been matched in terms of serum AMH levels and age. The aim of this study was first to identify risk factors for a poor ovarian response to hyperstimulation in women with OMA who were undergoing IVF/ICSI cycles, as compared to disease-free controls, and secondly to compare the ART outcomes.

## Materials and methods

### Study design

We conducted a cohort study that included the first IVF/ICSI cycle performed for each patient in the ART unit between 01/10/2012 and 31/12/2015 at the university-based reproductive medicine center of our institution. The study cohort was matched in terms of patient age and serum AMH levels. The women were allocated to two groups according to the study protocol: (i) a group made up of “exposed” women; i.e., who were afflicted with endometrioma (the ‘OMA group’) and (ii) an “unexposed” control group (the ‘control group’) that was comprised of women who did not exhibit endometriosis. Verbal non-opposition consent was obtained for each woman before inclusion in the database. All of the data were fully anonymized prior to their use. The study protocol was approved by the French Data Protection Authority (Commission Nationale de l’Informatique et des Libertés, CNIL) as approval n° 1988293 v 0).

### Patient cohort and the matching procedure

For both of the groups, the inclusion criteria for this cohort study were the following: a requirement for ART with in vitro fertilization (IVF) or intracytoplasmic sperm injection (ICSI), being less than 42 years of age at the time of the oocyte retrieval, and the first cycle having to be performed at our institution. Exclusion criteria were: a reliance on vitrified oocyte procedures and patients who had already been included in another ART research protocol.

All of the women with endometriosis-related infertility underwent an appropriate pre-ART work-up in order to obtain an accurate diagnosis and staging of their endometriosis [11]. For DIE and OMA phenotypes, the endometriosis diagnosis and staging were based on previously published imaging criteria using transvaginal ultrasonography (TVUS) [12–14], magnetic resonance imaging (MRI) [15–18], or transrectal ultrasonography [19]. More specifically, OMA was defined as a round-shaped cystic mass that had a minimum diameter of 1 cm, with thick walls, regular margins, a homogeneous low-echogenic fluid content with scattered internal echoes, and without papillary proliferations [20,21].

Women with a prior history of surgery for endometriosis were defined as patients who had previously undergone surgery for OMA, with or without exeresis of SUP and/or DIE lesions [22]. In addition, for the patients with a history of endometriosis surgery, the diagnosis was also confirmed by histological proof of the endometriosis. An associated adenomyosis was diagnosed based on TVUS and MRI imaging criteria [23].

Women with no signs of endometriosis were allocated to the control group [11].This group comprised women who were undergoing ART treatment for non-endometriosis-related infertility in the same time period as the case subjects. In these women, endometriosis was ruled out in a pre-ART work-up assessment, after a clinical examination, questioning (in regard to the extent of any pelvic pain and a prior history of surgery), and pelvic imaging (by TVUS and/or MRI).

Each OMA patient was matched to two disease-free women. The matching criteria were the following: patient age ± 1 year and serum AMH levels ± 0.2 ng/ml. Blind matching to the results was performed. Matching was performed by staff members who were cognizant of the matching criteria but who were otherwise blinded to the results. The matched records were used only once.

### IVF/ICSI procedures

The women were monitored and managed according to our institutional clinical protocols. All of the patients were synchronized using timed administration of an oral contraceptive (OC) containing 0.03 mg of ethinyl estradiol (EE) and 0.15 mg of levonorgestrel (LNG) (Minidril, Pfizer Holding, Paris, France), as described previously [24]. Various COS protocols were used, with 150–450 IU/day of recombinant FSH (Puregon-MSD, Courbevoie, France; Gonal-F, Merck, Lyon, France) and urinary FSH (hMG, Menopur, Ferring Pharmaceuticals, Gentilly, France), and comprised: (i) a Gonadotropin Releasing Hormone (GnRH) antagonist protocol, (ii) a long agonist protocol, or (iii) a short agonist protocol [25]. The gonadotropin doses and the COS protocol types were determined according to each patient’s individual characteristics [25]. The final oocyte maturation was triggered when ≥ 3 ovarian follicles that were ≥ 17 mm in diameter were visible by ultrasound, using either a single injection of 0.2 mg of GnRH agonist (triptoreline), or by injection of 250 μg of recombinant human chorionic gonadotropin (rHCG). The oocyte retrieval was performed 35–36 h later by transvaginal aspiration under ultrasound guidance. Embryo transfers (ET) were performed as either fresh or deferred [26], based on our institutional clinical protocols. The precise protocol of the embryo culture, cryopreservation, thawing, and transfer in our unit has been reported in detail previously [26].

### Data collection and outcome measures

All of the data were compiled in a computerized database (“Medifirst–Version 1.4.1“). The general characteristics of the patients in both of the groups were recorded prospectively, prior to the COS. The following data were collected: patient age; height; weight; body mass index (BMI); parity; gravidity; duration of their infertility; history of prior surgery for endometriosis and/or OMA; associated tubal and/or male factor of infertility; and ovarian reserve parameters (e.g. the AMH level and the antral follicle count (AFC)).

The primary outcome that was evaluated was the poor ovarian response (POR) rate. A POR to ovarian stimulation was defined as a cancelled cycle (following the development of less than three growing follicles) or the collection of ≤ 3 oocytes in response to the ovarian stimulation protocol [27,28].

The secondary outcomes that were evaluated included: the number of oocytes retrieved, the implantation rate (IR), the clinical pregnancy rate (cPR), the early miscarriage rate, the live birth rate (LBR) after the first embryo transfer, the cumulative pregnancy rate (cPR), and the cumulative live birth rate (cLBR).

The IR was defined as the number of gestational sacs observed divided by the number of embryos transferred [29]. Clinical pregnancy rates were determined by ultrasonographic documentation of at least one fetus with a heartbeat at 6–7 weeks of gestation [29]. Early pregnancy loss was defined as a spontaneous fetal demise at less than 10 weeks of gestational age [30]. The LBR was defined as delivery of a viable infant at 22 weeks or more of gestation [29]. The cumulative cPR and cLBR were defined as the proportion of women who received a transfer and who had at least one clinical pregnancy and live birth, respectively, whether from the first transfer attempt or subsequent transfers of frozen–thawed supernumerary embryos [31]. Once a woman obtained a live birth from IVF/ICSI she no longer contributed to the cumulative rates.

### Statistical analysis

The data were analyzed using IBM® SPSS® Statistics version 20.0 software (SPSS Inc. Headquarters, 233 S. Wacker Drive, 11^th^ floor, Chicago, Illinois 60606, USA). A *p-*value < 0.05 was considered to be statistically significant. Continuous data were presented as means and standard deviations; categorical data were presented as numbers and percentages. The patient characteristics and the ART outcome parameters were compared between the OMA and control groups by use of a Pearson’s Χ^2^ test or a Fisher’s exact test for qualitative variables and a Student’s t-test for quantitative variables, as appropriate.

A logistic regression analysis was performed to determine the variables that could be independently associated with a POR to stimulation. Confounding factors that were determined to be statistically significant at the threshold of p ≤ 0.10 by univariate analysis or with a clinical relevance were tested in a multiple logistic regression model. Interactions between explanatory variables were tested 2 by 2. Correlations between variables were tested, and if two variables were highly correlated, only one of them was introduced in the model. Age (> 35 years old (y.o.)), AMH levels (< 2 ng/ml), AFC (< 10), the total dose of injected gonadotropin, OMA status (categorized as follows: (i) No OMA; (ii) OMA without prior surgery for OMA; (iii) OMA with a prior history of surgery for OMA), the presence of OMA ≥ 2 cm in diameter, and the presence of bilateral OMAs were included in the multivariate analysis. Backward stepwise selection was used to retain variables with a *p*-value of < 0.05 in each final model. The parameter values for each of the final models were determined by the maximum likelihood method. Odds ratios (OR) and their 95% confidence intervals (95% CI) were calculated from the model’s coefficients and their standard deviations.

## Results

### Study population

**Fig 1:**
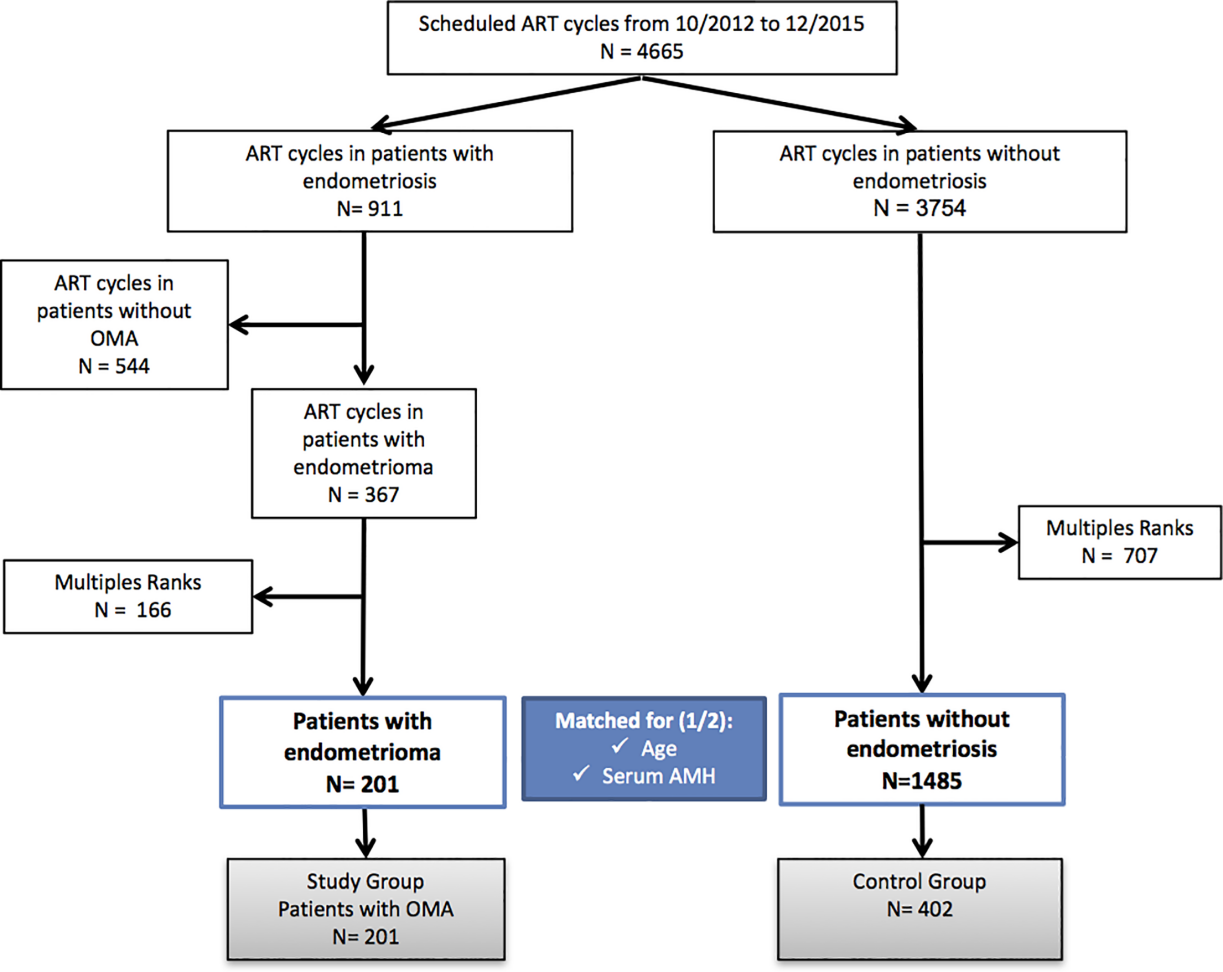
Patient inclusion flowchart. OMA: Ovarian endometrioma; ART: Assisted reproductive technology; AMH: anti-Müllerian hormone.

The process of our cohort selection is detailed in Fig 1. Overall, 201 patients with OMA (the OMA group) and 1,485 patients without endometriosis (the control group) were eligible for matching. These 201 women with OMA were matched by age and serum AMH level at a 2:1 ratio to the 401 endometriosis-free women. For the OMA group of patients, the specific endometriosis phenotype was as follows: 108 (53.7%) had unilateral OMA, while 93 (46.3%) had bilateral OMA. The mean size of the OMA lesions was 3.1 ± 2.1 cm for the right-hand side and 3.3 ± 2.2 cm for the left-hand side. An OMA diameter ≥ 2cm was found in 163/201 (81.1%) of these women. DIE was associated with the OMA in 100 (49.7%) of the women. Lastly, 48 (23.9%) of the patients were determined to have previously undergone surgery for OMA. Moreover, 73 (37.3%) of the women in the OMA group had an associated adenomyosis.

### Baseline characteristics

The baseline characteristics are presented in Table 1. As per the matching criteria, the serum AMH levels and the women’s ages were identical for both of the groups. The proportion of women with an AMH level lower than 1.1 ng/ml was not significantly different between the two groups (i.e., 35/201 (17.41%) in the OMA group and 73/402 (18.16%) in the control group; *p* = 0.823). However, the AFC was significantly lower for the OMA group compared to control group (i.e., 13.1 ± 7.4 versus 15.5 ± 10.1, respectively; *p* = 0.003) (Table 1).

**Table 1:** Baseline characteristics for the OMA group and the control group.

	OMA group n = 201	Control group n = 402	*p*-value
**Baseline characteristics**			
Age (years)*	33.7 ± 4.0	33.7 ± 4.0	NA
**Body Mass Index (kg/m^2^)**	22.9 ± 3.8	24.1 ± 4.7	0.001^a^
**Height (cm)**	170.0 ± 1	170.0 ± 1	0.145^a^
**Weight (kg)**	63.1 ± 11.7	65.5 ± 13.1	0.021^a^
**Smoking habits (n,%):**			0.431^b^
***Never smoked***	148 (73.6)	315 (78.4)	
***Current smoker***	32 (15.9)	53 (13.2)	
***Past smoker***	21 (10.5)	34 (8.4)	
**Type of infertility**			< 0.001^b^
***Primary***	161 (80.0)	283 (70.4)	
***Secondary***	40 (20.0)	119 (29.6)	
**Cause of the Infertility**			
***Associated with Male factor***	18 (9)	127 (31.6)	< 0.001^b^
***Associated with Tubal factor***	43 (21.3)	102 (25.3)	0.281^b^
**Patient’s ovarian reserve:**			
***AMH (ng/mL)*** *	3.4 ± 3.0	3.4 ± 3.0	NA
***AFC***	13.1 ± 7.4	15.5 ± 10.1	0.003^a^

AMH: Anti-Müllerian hormone; AFC: Antral follicle count. NA: not applicable

Data are the mean ± standard deviation or n (%), unless specified otherwise.

^a^ Student’s t-test.

^b^ Pearson’s χ2 test.

* As per matching criteria.

### Ovarian stimulation characteristics and ART outcomes

We mainly used an antagonist protocol with our cohort (i.e., 471/603 (78.1%) of the patients, amounting to 150/201 (75.1%) of the OMA group and 321/402 (80.1%) of the control group; *p* = 0.143). The duration of the stimulation was longer for the OMA group compared with the control group (i.e., 9.6 ± 1.7 days versus 9.2 ± 1.6 days, respectively; *p* = 0.006), and the total dose of gonadotropin that was used was higher for the OMA group compared with the control group (i.e., 2,602.0 ± 915.0 versus 2,353.0 ± 989.0, respectively; *p* = 0.003). The Day-8 estradiol levels for the OMA group were lower than for the control group (i.e., 845.6 ± 586.0 versus 1,027.0 ± 727.0, respectively; *p* = 0.005). The Day-8 progesterone levels did not differ between the two groups (i.e., they were 0.6 ± 0.4 versus 0.6 ± 0.3; *p* = 0.837for the OMA and the control group, respectively). The ovarian responsiveness was significantly lower in cases of OMA (Table 2): The rate of POR to ovarian stimulation was significantly higher for the OMA group (62/201 (30.8%) versus 90/402 (22.3%) in the control group; *p* = 0.02). The number of oocytes retrieved for the OMA group was significantly lower than for the control group (i.e., 7.5 ± 5.4 vs. 9.4 ± 6.1, respectively; *p* < 0.001), as was the number of mature oocytes in metaphase II (6.4 ± 4.8 vs. 7.6 ± 5, respectively; *p* = 0.009). However, the fertilization rates and the mean number of 2PN embryos obtained were not significantly different between the OMA and the control groups (Table 2).

**Table 2:** Ovarian stimulation characteristics and ART outcomes after the first ET for the OMA group and the control group.

	OMA group n = 201	Control group n = 402	*p*-value
**Number of oocytes retrieved**	7.5 ± 5.4	9.4 ± 6.1	< 0.001^a^
**Number of MII oocytes**	6.4 ± 4.8	7.6 ± 5.0	0.009^a^
**Number of 2PN embryos**	4.4 ± 3.4	4.9 ± 3.7	0.136^a^
**Maturation rate^c^**	0.9 ± 0.2	0.8 ± 0.2	0.029^a^
**Fertilization rate^d^**	0.7 ± 0.3	0.7 ± 0.3	0.138^a^
**Mean No. of blastocyst embryos obtained**	1.7 ± 3.1	2.1 ± 3.0	0.178^a^
Mean No. of embryos transferred *	1.5 ± 0.5	1.4 ± 0.5	0.143^a^
Implantation rate^e^*	0.3 ± 0.42	0.4 ± 0.48	0.102^a^
Clinical pregnancy rate *	53/151 (35.0)	134/324 (41.3)	0.226^b^
**Live birth rate** *	39/151 (25.8)	99/324 (30.5)	0.329^b^
Early pregnancy loss rate^f^*	14/53 (26.4)	32/134 (23.8)	0.851^b^

ART: assisted reproduction technology; ET: embryo transfer; OMA: endometrioma

Data are the mean ± standard deviation or n (%), unless specified otherwise.

^a^ Student’s t-test.

^b^ Pearson’s χ2 test.

^c^ maturation rate = the number of MII oocytes / the number of oocytes retrieved

^d^ fertilization rate = the number of 2PN / the number of mature oocytes

^e^ implantation rate = the number of gestational sacs /the number of embryos transferred

^f^ early pregnancy loss rate = the number of miscarriages / the number of clinical pregnancies

* After the first embryo transfer

An embryo transfer was achieved for 75.5% (151/201) of the women in the OMA group and for 80.5% (324/402) of the women in the control group (*p* = 0.121). After the first ET, in the OMA group, 81/151 (53.6%) of the women received a fresh embryo transfer (ET) and 70/151 (46.3%) received a deferred ET; in the control group, 228/324 (70.3%) of the women had a fresh ET and 96/324 (29.6%) had a deferred ET (p < 0.001). Similar proportions of Day-2 or blastocyst embryos were transferred in both groups (Day-2 ET and Blastocyst transfer: 120/151 (79.5%) and 31/151 (20.5%), respectively, for the OMA group versus 262/324 (80.8%) and 62/324 (19.2%), respectively, for the control group; *p* = 0.283).

The cumulative pregnancy rates [68/151 (45%) vs. 167/324 (51.5%), respectively; *p* = 0.19] and the cumulative live birth rates [47/151 (31.1%) versus 122/324 (37.6%), respectively; *p* = 0.16] were not significantly different between the OMA and the control groups.

A subgroup analysis was performed according to the characteristics of the OMA (i.e., an OMA diameter ≥ 2 cm and the presence of bilateral OMAs), and the results are shown in S1 Table. The ovarian stimulation and ART outcomes in our study were not significantly different in women with an OMA diameter ≥ 2cm as compared to those with a smaller OMA diameter (i.e., < 2 cm) and in women with bilateral as compared to unilateral OMA.

### Risk factors for a poor ovarian response to hyperstimulation

The results of the univariate analysis comparing the patients with a POR and those without are presented in Table 3. Age > 35 y.o. (OR = 2.1; 95% CI: 1.4–3.0; p < 0.001), AMH level < 2 ng/ml (OR = 3.8; 95% CI: 2.6–5.7; p < 0.001), AFC < 10 (OR:3.8; 95% CI: 2.5–5.6; p < 0.001), total dose of injected gonadotropin > 3,000 IU (OR = 1.6; 95% CI: 1.0–2.5; p = 0.021), and the presence of a prior surgery for OMA (OR = 2.4; 95% CI: 1.3–4.6; p = 0.004) were associated with a significantly higher risk of a POR. The presence of an associated DIE lesion was not found to be a risk factor for a POR.

**Table 3:** Logistic regression analysis of the risk factors for a poor response to hyperstimulation^#^.

	Univariate logistic regression analysis			Multiple logistic regression analysis ^a^		
Parameters	Odds ratio	95% CI^b^	*p-*value	Odds ratio	95% CI^b^	*p-*value
**Age at retrieval > 35 y.o.** *	2.1	1.4–3.0	< 0.001	1.7	1.1–2.5	0.014
**Body Mass Index (kg/m^2^) ≥ 30**	1.4	0.8–2.4	0.224			
**Smoking habits**						
***Past smoker versus never smoked***	0.8	0.4–1.4	0.488			
***Smoker versus never smoked***	0.8	0.4–1.7	0.714			
**AMH level < 2 ng/ml** *	3.8	2.6–5.7	< 0.001	2.6	1.7–4.0	< 0.001
**AFC < 10** *	3.8	2.5–5.6	< 0.001	2.4	1.6–3.7	< 0.001
**Type of infertility**						
***Secondary versus primary infertility***	1.0	0.6–1.6	0.788			
**Number of prior IVF/ICSI cycles**						
***One prior IVF/ICSI cycle versus no prior IVF/ICSI cycle***	0.8	0.3–1.7	0.566			
***Two or more prior IVF/ICSI cycles versus no prior IVF/ICSI cycle***	1.2	0.5–2.5	0.613			
**Type of embryo transfer strategy (Fresh versus deferred ET)**	1.0	0.6–1.8	0.981			
**Total dose of injected gonadotropin (IU)** *						
***≤ 1*,*500 IU*^c^**	0.4	0.2–0.8	0.008	1.3	0.7–2.4	0.425
***> 3*,*000 IU*^c^**	1.6	1.0–2.5	0.021	0.9	0.6–1.5	0.765
**OMA status** *						
***Presence of OMA and no prior surgery for OMA versus no OMA***	1.3	0.8–2.0	0.211	1.5	0.9–2.2	0.147
***Presence of OMA with a prior history of surgery for OMA versus no OMA***	2.4	1.3–4.6	0.004	2.1	1.1–4.0	0.033
**OMA associated with DIE Lesion**	1.0	0.6–1.8	0.962			
Presence of OMA ≥ 2cm in diameter*	1.4	0.9–2.1	0.096	0.8	0.4–1.9	0.643
Presence of bilateral OMAs*	1.3	0.8–2.2	0.238	1.1	0.6–2.2	0.737

y.o.: years old; AMH: Anti-Müllerian hormone; OMA: Endometrioma; DIE: Deep infiltrating endometriosis

^#^ poor response to hyperstimulation: ≤ 3 oocytes retrieved, or cycle cancelled

* variables included in the multiple logistic regression analysis

^a^ Age (> 35 y.o.), AMH levels (< 2ng/ml), AFC, Total dose of injected gonadotropin, OMA status, presence of OMA over 2 cm in diameter, and the presence of bilateral OMAs were included in the multiple logistic regression model.

^b^ 95% Confidence Interval

^c^ A total dose of gonadotropin comprised between 1,501 and 3,000 IU was considered as the reference.

After multivariate analysis, Age > 35 y.o. (OR = 1.7; 95% CI: 1.1–2.5; *p =* 0.015), AMH level < 2 ng/ml (OR = 2.6; 95% CI: 1.7–4.0; *p* < 0.001), AFC < 10 (OR = 2.4; 95% CI:1.6–3.7; *p* < 0.001), and OMA with a prior history of surgery for OMA (No OMA, OR = 1; OMA with a prior history of surgery for OMA, OR: 2.2, 95% CI: 1.1–4.2; *p* = 0.019) remained independent factors associated with an increased risk of a POR to hyperstimulation. In addition, OMA with no prior surgery for OMA, the presence of OMA ≥ 2cm in diameter, and the presence of bilateral OMAs were not significantly associated with an increased risk of a poor response (Table 3).

## Discussion

### Main findings

This age- and AMH-matched controlled study shows that the ovarian responsiveness to hyperstimulation was significantly reduced in women with OMA undergoing an IVF/ICSI cycle. Indeed, the rate of POR was significantly higher for the OMA group, and the number of oocytes retrieved was significantly lower, compared to the controls. However, the pregnancy and live birth rates were not reduced. Lastly, by multivariate logistic regression analysis our study showed that having previously undergone surgery for OMA was associated with an increased risk of a POR, as opposed to the presence of OMA without a prior history of surgery.

### Strengths and limitations

The strength of this study lies primarily in the methodological design: (i) Although previous series exploring the relationship between OMA and ovarian responsiveness to hyperstimulation have been reported in the literature, none of these studies performed a procedure to match for AMH levels. Our approach was designed to overcome potential bias related to the variation of the ovarian reserve in the evaluation of OMA responsiveness to the ART. Since the serum AMH level has been reported to be a robust biomarker of the ovarian response to gonadotropins, and as it has been shown to be strongly correlated with number of oocytes retrieved [32], evaluations of ovarian-responsiveness to hyperstimulation should be based on groups that are comparable in terms of serum AMH levels. In addition, as advanced age has a negative influence on ART outcomes [33,34] we also applied the age of the women as a matching criterion; (ii) The large number of OMA-affected patients that we enrolled (201 women) may have reduced selection bias and increased the statistical power of our study; (iii) Given the disease heterogeneity, we selected patients with well-defined endometriosis phenotypes (e.g., OMA with or without associated DIE) [35]. For OMA phenotypes, we only included patients for whom the diagnosis of endometriosis was based on stringent image-based criteria [12–14,18]. Additionally, for patients with a history of prior surgery for OMA, the diagnosis was also confirmed by histological proof; (iv) Lastly, numerous epidemiological variables were collected prospectively through face-to-face interviews before the ART procedure was performed (e.g., in regard to a prior surgical history, infertility data, and ovarian stimulation characteristics).

Despite the precautions that were taken, our study may nonetheless be subject to certain shortcomings and/or biases: (i) This study was conducted in a referral center for the management of endometriosis. Therefore, the women referred to our center may have been afflicted with particularly severe forms of endometriosis (e.g. 49.7% of the women in our study had a DIE phenotype). In addition, 23.9% of the patients had a prior history of surgery for OMA. This bias may have influenced the ovarian response to hyperstimulation. There is currently a growing consensus that surgical excision of OMA may damage the ovarian reserve and therefore limit the ovarian response to COS [36]. However, this bias would appear to be moderate given that, in light of the criteria that we employed for matching of the patient groups, the serum AMH levels were identical for both of the groups in our study. In addition, in our analysis, the presence of DIE associated lesions was not significantly associated with an increased risk of a poor response; (ii) Our study included women who were diagnosed with OMA through the use of imaging techniques. Our control group consisted of women with no clinical symptoms, or any radiological signs of endometriosis. It cannot be ruled out, however, that some women with asymptomatic superficial endometriosis were erroneously included in the disease-free group. We are aware that there is, in fact, no ideal control group for this type of endometriosis study [11]. Clearly though, the fact that our disease-free group may include a small number of women with asymptomatic SUP endometriosis does not lessen the clinical relevance of our finding that there was an increased risk of POR to hyperstimulation in the women with readily diagnosable OMA. (iii) We are cognizant that the type of embryo transfer (fresh or deferred ET strategy) differs between groups. Women in the control group had more often a fresh ET strategy than in the OMA group (p<0.001), but the type of transfer did not impact the ovarian response to stimulation (p = 0.981). In line with current scientific knowledge, the deferred ET strategy may increase pregnancy chances in endometriosis-affected women population [37]. However, the absence of randomized controlled studies in this population and the absence of superiority of this strategy in non-endometriosis women does not allow to differentiate between deferred ET and a fresh ET strategies [38,39]. In our study, the type of embryo transfer did not influence significantly pregnancy and live birth rates (data not shown).

### Interpretation

A systematic review by Hamdan *et al*. [4] has reported that, compared to disease-free women undergoing IVF/ICSI, women with OMA had a similar live birth rate, a lower mean number of oocytes retrieved, and a higher cycle cancellation rate. The potential impact of this OMA-related reduced responsiveness could be quantitative rather than qualitative. In addition, compared to women who had not undergone surgery, the authors did not observe differences regarding the number of oocytes retrieved or the cycle cancellation rate for women who had been surgically treated for OMA, although the AFC was lower. Unlike the study by Hamdan *et al*., most of the studies to date support the notion that OMA -and its excision- have an adverse effect on ovarian responsiveness, as they have found that IVF outcomes are significantly impaired in women operated on for OMA, and that the risk of a POR is higher in this population [40–45]. Our results suggest that OMA, and above all it surgical treatment, is responsible for a higher risk of a POR to hyperstimulation. By relying on a multivariate analysis to adjust for potentially confounding factors such as the women’s age, serum AMH levels, the dose of the injected gonadotropins, and the OMA status, we were able to identify factors related to a POR to hyperstimulation in our overall population. While OMA may exert a detrimental impact on the number of oocytes retrieved, we found that OMA *per se* was not associated with a poor ovarian response to hyperstimulation. Conversely, a prior history of OMA surgery had a significant negative impact on the POR to stimulation (OR 2.1, 95% CI: 1.1–4.0). This factor is an independent predictive factor of a poor ovarian response. Potential deleterious mechanisms of surgery comprise the accidental removal of healthy ovarian tissue during cystectomy, as well as the surgery-related local fibrosis or vascular impairment following electrosurgical coagulation [46,47]. Thus, there is increasing evidence supporting the notion that ovarian cystectomy is detrimental to the ovarian reserve [46,48,49].

The serum AMH level has been shown to be a good predictor of the ovarian response to COS [50–52]. In order to analyze the impact of endometriotic cysts on the ovarian response and whether surgery could influence the risk of a POR, ovarian reserve parameters such as the serum AMH level must be taken into account. However, none of the studies to date have taken into account the AMH level. By applying a matching procedure in this study for serum AMH levels, we sought to overcome potential bias related to variation of the ovarian reserve in the evaluation of ovarian responsiveness to ART in women with OMA. In a recent study, Nelson *et al*. showed that the serum AMH level was a better predictor of the ovarian response to hyperstimulation than the AFC, in both randomized trials utilizing GnRH agonist and GnRH antagonist protocols [32]. Furthermore, large multicenter trials [53,54] have shown that the AFC is a worse predictor of the ovarian response to COS than the serum AMH level, and that the AFC provides no additional predictive value above and beyond serum AMH levels. Moreover, in clinical practice, measurement of the AFC is known to exhibit substantial intra- and inter-operator variability [55]. Lima *et al*. showed that in women with endometriosis that the AFC might be underestimated in the presence of OMA [56]. This fact could be secondary to an impaired ability to detect small follicles in the presence of an OMA. On the other hand, Benaglia *et al*. found a similar accuracy for predicting the ovarian response with the AFC in unaffected ovaries, ovaries with endometriomas, and ovaries with a history of surgery for endometriomas [57]. Nevertheless, the answer to the question as to whether the serum AMH level or the AFC is the best ovarian responsiveness marker for women with OMA is still a matter of considerable debate. In our AMH-matched study, the AFC was found to be lower in women with OMA, as was the ovarian responsiveness to hyperstimulation.

Our findings have clinical implications that are of relevance to daily practice. First, our study confirms that OMA could negatively influence ovarian responsiveness in terms of quantity but not in terms of quality, since the live birth rates were similar for the women with OMA as compared to their disease-free counterparts. Damage secondary to OMA *per se* does not appear to be sufficient to influence oocyte quality. However, given the impact of OMA surgery on the ovarian response to hyperstimulation, it would seem appropriate to as much as possible provide infertile women with the option of IVF/ICSI, in accordance with the intensity of their pain symptoms and their wishes. For women who do not intend to become pregnant in the near future but who are scheduled to undergo surgery for OMA, fertility preservation could be a suitable option prior to the surgery in order to limit the risk of a POR after the surgical treatment. Our results therefore support the notion that fertility preservation should be part of the preoperative advice that is provided to young women with severe endometriosis [58].

## Conclusion

Our findings indicate that the presence of OMA at the time of ART increases the risk of a POR to hyperstimulation, although OMA does not appear to negatively affect the pregnancy rate. Furthermore, we found that a prior history of surgery for OMA significantly increases this risk. These findings may be applicable in daily clinical practice by contributing to the optimization of the management of infertile patients with OMA.

## Supporting information

S1 Table Baseline characteristics, COS, and ART outcomes in women exhibiting bilateral OMAs or an OMA with a diameter greater than 2 cm. (DOCX)
