# Supplementary material for: Endometriosis and ART: A prior history of surgery for OMA is associated with a poor ovarian response to hyperstimulation
Source: PLoS One. 2018 Aug 20;13(8):e0202399. doi: 10.1371/journal.pone.0202399 (PMC6101383; doi:10.1371/journal.pone.0202399)
Supplement: S1 Table — (DOCX) [file pone.0202399.s001.docx]

|  | OMA < 2 cm  n = 38 | OMA ≥ 2 cm  n = 163 | Unilateral OMA  n = 108 | Bilateral OMA  n = 93 |
| --- | --- | --- | --- | --- |
| Age (years) | 32.5 ± 3.7 | 33.96 ± 4.02 ^a^ | 34.1 ± 4.1 | 33.2 ± 3.8 |
| Body Mass Index (kg/m^2^) | 23.1 ± 3.9 | 22.7 ± 3.8 | 22.9 ±4.0 | 22.8 ± 3.7 |
| Smoking habits |  |  |  |  |
| Never smoked | 26 (68.4) | 122 (74.8)) | 77 (71.3) | 71 (76.3) |
| Current smoker | 9 (23.7) | 23 (14.1) | 22 (20.4) | 10 (10.8) |
| Past smoker | 3 (7.9) | 18 (11.0) | 9 (8.3) | 12 (12.9) |
| Type of infertility |  |  |  |  |
| Primary | 27 (71.1) | 134 (82.7) | 82 (75.9) | 79 (84.9) |
| Secondary | 11 (28.9) | 29 (17.3) | 26 (24.1) | 14 (15.1) |
| Cause of the Infertility |  |  |  |  |
| Associated with Male factor | 3 (7.9) | 15(9.2) | 11 (10.2) | 7 (7.5) |
| Associated with Tubal factor | 6(15.8) | 37(22.7) | 28(13.0) | 15 (15.1) |
| Patient’s ovarian reserve: |  |  |  |  |
| AMH (ng/mL) | 3.0 ± 2.3 | 3.5 ± 3.1 | 3.5 ± 3.2 | 3.3 ± 2.7 |
| AFC | 12.8 ± 7.7 | 13.1 ± 7.3 | 13.5 ± 7.3 | 12.5 ± 7.5 |
| Number of oocytes retrieved | 7.1 ± 4.6 | 7.5 ± 5.5 | 7.0 ±5.6 | 8.0 ± 5.2 |
| Number of MII oocytes | 6.2 ± 4.1 | 6.5 ± 4.9 | 6.0 ± 4.7 | 7.0 ± 4.8 |
| Number of 2PN embryos | 4.4 ± 3.2 | 4.4 ± 3.5 | 4.0 ± 3.5 | 4.9 ± 3.4 |
| Maturation rate ^b^ | 0.9 ±0.1 | 0.9 ± 0.2 | 0.9 ± 0.2 | 0.9 ±0.2 |
| Fertilization rate ^c^ | 0.7 ± 0.3 | 0.7 ± 0.3 | 0.7 ±0.3 | 0.7 ±0.4 |
| Number of blastocyst embryos obtained | 1.9 ± 3.4 | 1.7 ± 3.1 | 1.5 ± 3.2 | 2.0 ± 3.0 |
| Rate of poor ovarian response to stimulation | 13 (34.2) | 49 (30.1) | 34 (31.5) | 28 (30.1) |
| Mean No. of embryos transferred * | 1.5 ± 0.5 | 1.5 ± 0.5 | 1.5 ±0.5 | 1.5 ± 0.5 |
| Implantation rate ^d^ * | 0.3 ± 0.4 | 0.3 ± 0.4 | 0.3 ± 0.4 | 0.2 ±0.4 |
| Clinical pregnancy rate * | 10/27 (37.0) | 43/124 (34.7) | 34/82 (41.5) | 19/69 (27.5) |
| Live birth rate * | 7/27 (25.9) | 32/124 (25.8) | 22/82 (26.8) | 17/69 (24.6) |
| Early pregnancy loss rate^e^ * | 3/10 (30.0) | 11/43 (25.6) | 11/34 (32.4) | 2/19 (10.5) |
| Cumulative live birth rate | 12/27 (44.4) | 35/124 (28.2) | 27/82 (32.9) | 20/69 (29.0) |

S1 Table. Baseline characteristics, COS, and ART outcomes in women exhibiting bilateral OMAs or an OMA diameter greater than 2 cm

COS: controlled ovarian stimulation; ART: assisted reproduction technology; ET: embryo transfer; OMA: Endometrioma; AMH: Anti-Müllerian hormone; AFC: Antral follicle count; MII: metaphase 2 oocytes; 2PN: 2 Pronuclei.

Data are the mean ± standard deviation or n (%), unless specified otherwise.

* After the first embryo transfer

a Same superscript letter in a row indicate a statistically significant difference (p < 0.05) using the Student’s t-test or the Pearson’s χ2 test, as appropriate.

^b^ maturation rate= the number of MII oocytes / the number of oocytes retrieved

^c^ fertilization rate=the number of 2PN / the number of mature oocytes

^d^ implantation rate = the number of gestational sacs / the number of embryos transferred

^e^ early pregnancy loss rate = the number of miscarriages /the number of clinical pregnancies
